# Microbial Degradation of Aldrin and Dieldrin: Mechanisms and Biochemical Pathways

**DOI:** 10.3389/fmicb.2022.713375

**Published:** 2022-03-29

**Authors:** Shimei Pang, Ziqiu Lin, Jiayi Li, Yuming Zhang, Sandhya Mishra, Pankaj Bhatt, Shaohua Chen

**Affiliations:** ^1^State Key Laboratory for Conservation and Utilization of Subtropical Agro-bioresources, Guangdong Provincial Key Laboratory of Agricultural and Rural Pollution Abatement and Environmental Safety, Integrative Microbiology Research Centre, South China Agricultural University, Guangzhou, China; ^2^Guangdong Laboratory for Lingnan Modern Agriculture, Guangzhou, China; ^3^Environmental Technologies Division, CSIR-National Botanical Research Institute, Rana Pratap Marg, Lucknow, India

**Keywords:** aldrin, dieldrin, environmental fate, toxicity, microbial degradation, degradation mechanisms

## Abstract

As members of the organochlorine group of insecticides, aldrin and dieldrin are effective at protecting agriculture from insect pests. However, because of excessive use and a long half-life, they have contributed to the major pollution of the water/soil environments. Aldrin and dieldrin have been reported to be highly toxic to humans and other non-target organisms, and so their use has gradually been banned worldwide. Various methods have been tried to remove them from the environment, including xenon lamps, combustion, ion conversion, and microbial degradation. Microbial degradation is considered the most promising treatment method because of its advantages of economy, environmental protection, and convenience. To date, a few aldrin/dieldrin-degrading microorganisms have been isolated and identified, including *Pseudomonas fluorescens*, *Trichoderma viride*, *Pleurotus ostreatus*, *Mucor racemosus*, *Burkholderia* sp., *Cupriavidus* sp., *Pseudonocardia* sp., and a community of anaerobic microorganisms. Many aldrin/dieldrin resistance genes have been identified from insects and microorganisms, such as *Rdl*, *bph*, *HCo-LGC-38*, *S2-RDL^A302S^*, *CSRDL1A*, *CSRDL2S*, *HaRdl-1*, and *HaRdl-2*. Aldrin degradation includes three pathways: the oxidation pathway, the reduction pathway, and the hydroxylation pathway, with dieldrin as a major metabolite. Degradation of dieldrin includes four pathways: oxidation, reduction, hydroxylation, and hydrolysis, with 9-hydroxydieldrin and dihydroxydieldrin as major products. Many studies have investigated the toxicity and degradation of aldrin/dieldrin. However, few reviews have focused on the microbial degradation and biochemical mechanisms of aldrin/dieldrin. In this review paper, the microbial degradation and degradation mechanisms of aldrin/dieldrin are summarized in order to provide a theoretical and practical basis for the bioremediation of aldrin/dieldrin-polluted environment.

## Introduction

Due to the largescale exploitation of natural resources and the destruction of the natural environment by human beings, the pressure on the biosphere is increasing (Akhtar and Mannan, 2020). The main causes of ecological pollution are excessive mining activities, industry, waste disposal, and agrochemicals (Gupta et al., 2020). Persistent organic pollutants (POPs) are toxic synthetic chemicals impacting on the environment, and have become a hot topic. POPs can accumulate in organisms and act as endocrine disruptors or carcinogens. Although the government has banned the use of POPs, the harm caused by these pollutants to the environment has continued because of people’s misuse of them and the long half-life of POPs (Adithya et al., 2021). It is, therefore, urgent to find a safe and effective method to avoid long-term damage to the environment.

Aldrin (CAS 309-00-2) and dieldrin (CAS 60-57-1) are synthetic organochlorine cyclodiene pesticides used to control subterranean insect pests such as nargles root maggots, mole cricket grubs and weevils, in agriculture. Aldrin’s (1,2,3,4,10,10-Hexachloro-1,4,4α,5,8,8α-hexahydro-1,4-endo, exo-5,8-dimethanonaphthalene) molecular formula is C_12_H_8_C_*l*6_ and its half-life in soil is estimated to be 1.5–5.2 years (Blaylock, 2005a,b). Dieldrin’s (1,2,3,4,10,10-Hexachloro-6,7-epoxy-1,4,4α,5,6,7,8,8α-octahydro-1,4-endo, exo-5,8-dimethanonaphthalene) molecular formula is C_12_H_8_C_*l*6_O and its half-life in soil is approximately 5 years (Blaylock, 2005b). In the natural environment, aldrin is usually converted to dieldrin by biotic or abiotic mechanisms, and the half-life of dieldrin is significantly longer. This review discusses dieldrin and its degradation products aldrin together because of their similar structure and properties (Blaylock, 2005a,b). They were widely used in the late 1940s; however, due to their toxicity and accumulation in the food chain, they were gradually banned in developed and developing countries in the late 1970s and 1980s (Luzardo et al., 2006). Organochlorine pesticides (OCPs) are major POPs, which have been banned or restricted worldwide, including aldrin and dieldrin (Adeleye et al., 2019).

Although reports of resistance to the cyclodiene pesticides aldrin and dieldrin account for more than 60% of reported cases of resistance, the overall incidence of related cases of resistance are declining (Ffrench-Constant et al., 2000). Therefore, various countries have launched the monitoring of organochlorine pesticides (Tham et al., 2020). Developed countries banned them early on, so most of the reported toxicity incidents involving aldrin and dieldrin now come from developing countries. Pesticide contamination is more severe in poorer countries, and its negative effects on ecology and life are therefore more profound (Høyer et al., 2000). The degradation of POPs includes physical and chemical degradation and biodegradation. Physical, chemical, and biological factors in the natural environment promote the degradation of POPs, of which biological factors play the major role (Chen et al., 2013; Lin et al., 2020; Pang et al., 2020b). Microorganisms are among the most important biological factors, including bacteria, fungi, and algae (Boudh et al., 2019; Lin et al., 2020). Physical and chemical methods, such as electro-oxidation, incineration, microwave induction, and chemical oxidation, not only require large and expensive infrastructure, but also produce byproducts that cannot be completely degraded and sometimes are even more toxic than the parent compounds (Pang et al., 2020a; Zhang et al., 2020). Microbial degradation, as one of the traditional treatment methods, is more economical and environmentally friendly because it degrades more completely (Zhang et al., 2021). As a result of the extensive use of aldrin and dieldrin, many non-microbial resistance genes have been detected in many places. The researchers isolated many strains that were able to effectively degrade aldrin and dieldrin (Abbas et al., 2020). The carbon component is an important driver of microbial community and function. A large number of genomic and complex metabolic studies have confirmed that dieldrin degradation evolved in a low-carbon environment, which provides a good direction to further isolate degrading microorganisms (Krohn et al., 2021).

Aldrin and dieldrin have been used for more than 80 years, and a lot of research has been conducted to investigate their toxicity and degradation (Matsumoto et al., 2008; Birolli et al., 2015; Wang et al., 2020). However, few reviews have focused on the microbial degradation and degradation mechanisms of aldrin/dieldrin. In this review, the residues of aldrin/dieldrin and their toxic effects on non-target organisms are discussed. Moreover, the microbial strains and their degradation enzymes/genes for aldrin/dieldrin that are responsible for biodegradation are summarized. This review updates the knowledge on degradation strategies with a focus on the metabolic pathways and molecular mechanisms involved in the biodegradation of aldrin/dieldrin.

## Toxicity of Aldrin and Dieldrin

**FIGURE 1:**
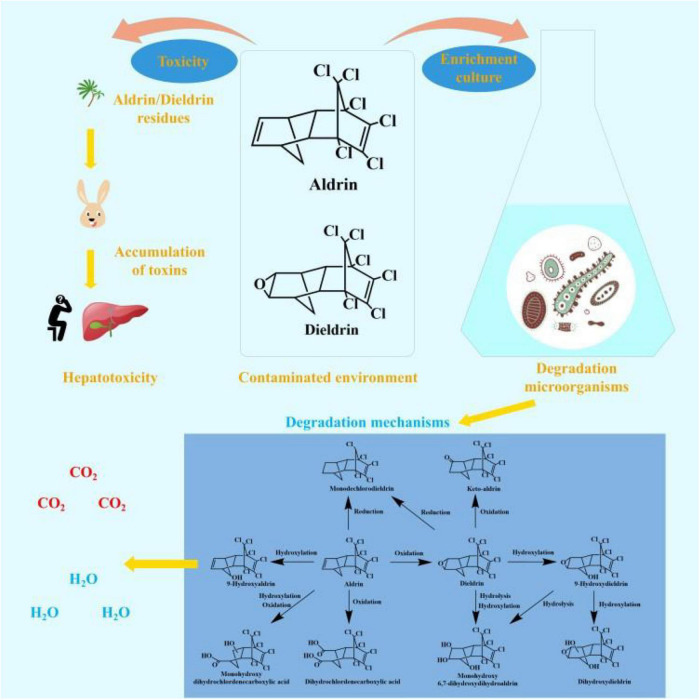
The toxicity and environmental fate of aldrin and dieldrin.

Aldrin and dieldrin belong to the same group of pesticides, most of which have the same toxicology (Hidayati et al., 2021). They have been in use for more than 80 years and have had a profound impact on the world (Hua et al., 2021). Although these two pesticides are banned around the world, concentrations can still be detected in some places. The remaining aldrin and dieldrin in the environment have a persistent toxic effect on local ecosystems and non-target organisms. The toxicity and environmental fate of aldrin and dieldrin is shown in Figure 1.

### Environmental Accumulation of Aldrin and Dieldrin

During the use of pesticides, only 10% of their volume is utilized, while the other 90% will remain in the ecosystem (Lin et al., 2022). The pesticide residues migrate to other places *via* volatilization, washing, dust, etc., which in turn causes wider pollution (Bhatt et al., 2021; Mishra et al., 2022). Here is a systematic summary of aldrin and dieldrin contamination reported in different places in recent years. A combination of gas chromatography and electron capture detection (GC-ECD) was used to determine organochlorine pesticides in two common leafy vegetables from southwest Nigeria. Risk assessments indicated that aldrin and dieldrin may pose a carcinogenic health risk to adults (60 kg) and children (16.7 kg) (Adeleye et al., 2019). The concentrations of dieldrin detected in surface and ground water consumed in the cacao crop-dominated agricultural catchment of Ancobra Basin, Ghana, may lead to cancer in local children (Affum et al., 2018). Surface water from the Thamirabarani River water system in southern India was detected at aldrin levels above the World Health Organization’s maximum residue limit for surface water (0.03 g⋅L^–1^) (Arisekar et al., 2019). A health risk assessment of fish in Lake Saint Lucia indicated that residual concentrations of aldrin and dieldrin in fish muscle tissue could lead to potential dietary risks in humans (Buah-Kwofie and Humphries, 2021). The estimated daily intake of aldrin or dieldrin was found to exceed the allowable daily intake in breastmilk from urban and semi-urban areas and was greater in urban than in semi-urban areas (Anand et al., 2021). An investigation of dieldrin exposure in 8-year-old Latinx boys and girls from rural, farm-worker families and urban, non-farm-worker families in North Carolina found that dieldrin was pervasive in the living environments of children in vulnerable immigrant communities (Arcury et al., 2021). Estimated daily intake values (EDI) of aldrin and dieldrin by cattle slaughtered in Benin, southern Nigeria exceeded the threshold, leading to a non-cancer health risk for children consuming contaminated cattle parts (Tongo and Ezemonye, 2015).

With emphasis on the ecological environment, countries around the world have reported on their own environmental pollution. Rwanda’s first assessment of contaminants in its soil found levels of aldrin in the range of 0.38–0.59 μg⋅kg^–1^⋅dw (Umulisa et al., 2020). A decline in dieldrin concentrations in the Niagara River was detected using a Hilbert–Huang transform analysis (Tsai and Treadwell, 2019). Dieldrin has been found in water, fish, sediments, and large parasites in Okobaba, Lagos Lagoon, Nigeria (Bamidele et al., 2019). High concentrations of polyaromatic hydrocarbons (PAHs) and polychlorinated biphenyl (PCBs) were found in samples collected from coastal areas in the refinery area of the Kingdom of Bahrain (Bersuder et al., 2020). An organochlorine pesticide analysis of shrimp from all continents found that Asia was the continent with the highest reported values, with aldrin and dieldrin concentrations of 0.003 μg⋅L^–1^ (Maia et al., 2020). The concentrations of dieldrin in the water, soil, and sediment of the Ruffiji River Delta in Tanzania exceeded safety guidelines, with dire potential consequences if they are not managed and emergency interventions taken (Mwevura et al., 2021). The highest concentrations of dieldrin (0.99 ± 0.33 μg⋅kg^–1^) were found in the sediments of southern and southern river ecosystems in Nigeria (Ogbeide et al., 2019). Surveillance of pesticides in rivers and lakes in northern Greece found average annual concentrations of dieldrin (0.02 μg⋅L^–1^) exceeding the EU environmental quality standards (Papadakis et al., 2015). Concentrations of organochlorine pesticides detected in Serbian river and artificial lake sediments ranged from below the detection limit to 113 μg⋅kg^–1^ (Sakan et al., 2017). Aldrin (75.31 ng⋅L^–1^) and dieldrin (71.19 ng⋅L^–1^) were found in stream water in Mumbai, India, exceeding their respective standard levels but below the range set by the America Environmental Protection Agency (EPA) at 0.13 65.1 ng⋅L^–1^ (Singare, 2016).

### Toxicity of Aldrin and Dieldrin in Living Systems

Aldrin and dieldrin pose a risk to human health throughout the world because they accumulate in the food chain and the human body cannot metabolize them (Maia et al., 2020). Dieldrin is immunogenic to humans, leading to dopaminergic neurodegeneration, which causes chemically immunohemolytic anemia or gives rise to Parkinson’s disease (Hamilton et al., 1978; Kanthasamy et al., 2005, 2020). Contact with dieldrin can induce the production of reactive oxygen species or cause proinflammatory reaction and DNA damage in human ovary surface epithelial cells (Shah et al., 2020). Aldrin acts as a ligand for androgens and alters epigenetic inheritance, which may contribute to prostate cancer development and growth. Exposure to dieldrin causes an aggravation of the behavioral and biochemical deficits induced by male-specific synuclein disease (Gezer et al., 2020). Dieldrin can induce estrogen action and inhibit androgen signaling pathways, which can lead to breast cancer (Aubé et al., 2011).

In mammals, as in humans, aldrin and dieldrin accumulate toxicity through the food chain. Dieldrin inhibited the activity of Mg^2+^-ATPase in rats and stimulated the activity of 5′-nucleotide enzyme and NADH-dehydrogenase in liver cell membranes, and L-ascorbic acid was needed to protect the Mg^2+^-ATPase (Bandyopadhyay et al., 1982). When rats were exposed to aldrin over a period of time, the relative liver weight increased significantly, and the survival rate of female rats decreased (Adam et al., 2015). In Punjab, India, bioaccumulation of 14 pesticides, including dieldrin and aldrin, has been found in the tissue stroma of dogs with malignant canine breast tumors (Gautam et al., 2020).

### Toxicity of Aldrin and Dieldrin in Aquatic Organisms

The biodegradable potential of pesticides can have a significant impact on the population dynamics of aquatic species. In ecological model assessments, the population of aquatic species changes when pesticides remain in the water for long periods of time (Achema et al., 2021). Among non-target aquatic organisms, pesticides have the greatest negative impact on fish, followed by water fleas and algae (Affum et al., 2018). Endocrine reactions in fish are related to the pollutant load in sediments. In Lagos Lagoon, Nigeria, contaminated with dieldrin, male fish gradually feminized, while the reproductive health of other fish also changed accordingly (Adeogun et al., 2019). Dieldrin inhibited the liver cytochrome P-450 expression of female *Micropterus salmoides* during the breeding period (Barber et al., 2007). Aldrin/dieldrin is the contaminant most associated with oxidative damage in the livers of two wild fish species at the Furnas Hydroelectric Power Station (HPS) reservoir (Minas Gerais, Brazil) (Sakuragui et al., 2013). After 24 h exposure, dieldrin significantly affected *Danio rerio* and *Daphnia pulex*’s swimming behavioral response and sensitivity (Alla et al., 2021). As a teratogen, dieldrin can alter the expression of dopamine receptor 2A and dopamine transporter in zebrafish (*Danio rerio*) embryos (Sarty et al., 2017).

## Degradation of Aldrin and Dieldrin

In the natural environment, aldrin and dieldrin remain in the air, soil, and water. The physical and chemical conditions directly affect the behavior and distribution of aldrin and dieldrin residues. During their interaction with organic or mineral components in the soil, natural degradation occurs. Due to the indiscriminate use of organic pollutants, which hardly degrade under natural conditions, many methods have been developed to facilitate their degradation, including physicochemical methods, the use of microorganisms, and gene therapy (Feng et al., 2020).

### Degradation of Aldrin and Dieldrin by Physicochemical Methods

Under the irradiation of a xenon lamp below 290 nm, aldrin reacts with α-diketones such as glyoxal, phenylglyoxal, methyl glyoxal, diacetyl, 1-phenyl-1, 2-malondione, and phenyl to form the corresponding epoxide compound, dieldrin (Nojima and Isogami, 1993). After gas phase thermal decomposition and toxic combustion, the pyrolysis of dieldrin yields chlorinated benzene and chlorinated phenols, known as precursors of polychlorinated dibenzo-p-dioxins and dibenzo-furans (Dharmarathne et al., 2019). The non-pathogenic bacterium *Shevanella oneidensis* does not directly degrade dieldrin, but can efficiently reduce about 80% of dissolved ferric iron (Fe^3+^) to ferrous iron (Fe^2+^) within 72 h. The effective removal time of many organochlorine pesticides (OCPs) is greatly reduced by the catalysis of ferrous iron (Fe^2+^). When the iron dosage was increased from 2.5 × 10^3^ mg⋅L^–1^ to 1.5 × 10^4^ mg⋅L^–1^, the removal rate of dieldrin was increased by 23.3% (Abbas et al., 2020). Physicochemical reactions are rarely used in real life because they cannot completely degrade compounds and are limited to large instruments (Lin et al., 2020).

### Degradation of Aldrin and Dieldrin by Microorganisms

Bioremediation of aldrin and dieldrin is possible with the application of indigenous microbial strains (Arora, 2020; Bhatt et al., 2022). This requires effective microbial food and nutrients, known as the bioremediation triangle (Boudh et al., 2019). Microorganisms take pollutants as their main carbon and nitrogen sources or auxiliary nutrients to grow and consume pollutants so that they achieve the purpose of removing pollutants (Bourtzis et al., 2016; Cycoń et al., 2017; Mishra et al., 2021). In the development of bioremediation methods, it is very important to screen microorganisms that degrade organic pollutants and identify their metabolites. Bacteria, fungi and algae are used in biodegradation experiments (Zhao et al., 2017, 2020; Bhatt et al., 2020; Huang et al., 2020). Yeast is a common eukaryotic recipient cell for gene cloning experiments (Chen et al., 2012; Ali et al., 2020). Actinomycetes are particularly suitable for the colonization of terrestrial ecosystems because of their mycelial growth pattern and ability to produce large amounts of extracellular enzymes (Chen et al., 2011; Magan et al., 2022). However, there are no available reports on the ability of yeasts and actinomycetes to degrade aldrin/dieldrin. Till date, there are several bacteria and fungi that have been clearly reported to effectively degrade aldrin/dieldrin (Sakakibara et al., 2011; Xiao et al., 2011a; Purnomo et al., 2017). In addition, the degradation of a microbial consortium has been studied (Watanabe and Yoshikawa, 2008). Aldrin and dieldrin are degraded in the soil because a few microorganisms can efficiently metabolize them, as shown in Table 1.

**TABLE 1:** Microbial degradation of aldrin/dieldrin.

Classification	Strains	Sources	Comments	References
Bacteria	*Pseudomonas fluorescens*	No data	77.3% of 10 mg⋅L^–1^ dieldrin and 94.8% of 10 mg⋅L^–1^ aldrin were degraded within 120 h.	Bandala et al., 2006
	*Burkholderia* sp. Med-7 and *Cupriavidus* sp. Med-5	Agricultural fields	They can degrade 49 and 38% of dieldrin, respectively.	Matsumoto et al., 2008
	*Enterobacter* sp. LY402	No data	Degradation of 40.4% of dieldrin (5.0 mg⋅L^–1^) in 168 h	Gao et al., 2008
	*Pseudonocardia* sp. KSF27	Agricultural sites	Approximately 85% of 14.06 μM of dieldrin was degraded within 5 days	Sakakibara et al., 2011
Fungi	*Trichoderma viride*	Dieldrin- treated soil	They contribute to the high degradation of dieldrin	Matsumura and Bous, 1967
	*Mucor alternans*	Agricultural loam Soil	It can degrade dieldrin in the laboratory, but not in the natural environment.	Zhao et al., 2017
	*Phanerochaete chrysosporium* and *Trametes versicolor*	No data	They can completely degrade 5–30 mg⋅L^–1^ of dieldrin within 25 days.	Fragoeiro and Magan, 2005
	Wood-rotting fungi strain yk543	Rotten wood	It degraded approximate 39.1% of dieldrin within 30 days.	Kamei et al., 2010
	*Mucor racemosus* ddf	Agricultural Sites	It degraded 95.8% of 13.2 μM of dieldrin within 20 days.	Kataoka et al., 2010
	*Phlebiaacanthocystis*, *Phlebia brevispora*, and *Phlebia aurea*	Tottori Mycological Institute	They can degrade over 50% of dieldrin within 42 days and 90% of aldrin within 28 days, respectively.	Xiao et al., 2011a
	*Pleurotus ostreatus*	Collection of Lab, Kyushu University, Japan	It degraded 100% of aldrin and 18% of dieldrin in 14 days.	Purnomo et al., 2017
	*Penicillium miczynskii* CBMAI 930	Marine	Converted up to 90% (50 mg⋅L^–1^) of dieldrin within 14 days.	Birolli et al., 2015
Microbial consortium	Mixed microorganisms including *Clostridium bifermrntans*, *Clostridirum glwolium*, and *Clostridium* sp.	Freshwater mud	96% of 10 mg⋅L^–1^ dieldrin was degraded within 7 days	Maule et al., 1987
	Methanogenic granular sludge	Sludge	88% of dieldrin (9 mg⋅L^–1^) was transformed within 3 months; 70% of aldrin (7 mg⋅L^–1^) was transformed within 160 days.	Baczynski et al., 2004
	Mixed indigenous microorganisms including *Acidaminobacter*, *Clostridium* and an uncultured bacterial group	Anaerobic Sediment	Degrading low concentrations of dieldrin (from 0.5 to 10 μg⋅mL^–1^)	Chiu et al., 2005
	Community of 11 morphologically identical anaerobic microorganisms	Paddy field soil	They can degrade up to 75.6% of dieldrin (100 mg⋅L^–1^), and 65.4% of aldrin (100 mg⋅L^–1^), respectively, within 2 weeks.	Watanabe and Yoshikawa, 2008

#### Bacterial Degradation of Aldrin/Dieldrin

*Pseudomonas* is effective at degrading a variety of pesticides in water, such as dieldrin, aldrin, and heptachloride (Bandala et al., 2006; Birolli et al., 2019). However, due to the low bioavailability of dieldrin, it is difficult to isolate more degrading microorganisms. Matsumoto et al. (2008) obtained *Burkholderia* sp. Med-7 and *Cupriavidus* sp. Med-5 degrading bacteria with dieldrin as the only carbon source by screening bacteria with 1,2-epoxycyclohexane (ECH), which can degrade 49 and 38% of dieldrin, respectively. *Enterobacter* sp. LY402 is able to degrade 40.4% of dieldrin (5.0 mg⋅L^–1^) in 168 h (Gao et al., 2008). In addition, the effective strain *Pseudonocardia* sp. KSF27 was successfully isolated by using a soil–charcoal perfusion method with aldrin transdiol as an analog substrate (Sakakibara et al., 2011). The bioremediation potential of this strain is very high. It not only degrades 85% of 14.06 μM of dieldrin, but also degrades a variety of refractory pollutants, such as endosulfan, endosulfan sulfate, and heptachloride.

The above test results also prove that aldrin transdiol and ECH are effective structural analogs that can be used to isolate degrading microorganisms capable of degrading aldrin/dieldrin. This is also a good guideline that could be used to screen the degradation microorganisms of other hard-to-degrade compounds. The use of structural analogs is considered a useful method for isolating microorganisms with highly refractory compounds, and to be less time-consuming and labor-saving than conventional methods. In addition, co-metabolism plays an important role in the process of bioremediation and is an important direction for screening degrading bacteria (Matsumoto et al., 2008).

#### Fungal Degradation of Aldrin/Dieldrin

Fungi are generally more tolerant to high concentrations of contaminant chemicals than bacteria, and white rot fungi (WRF) have been especially well studied. In previous studies, WRF was able to oxidize a wider variety of refractory compounds, such as lindane, lignin, dichlorodiphenyltrichloroethane (DDT) (Xiao et al., 2011b; Hou et al., 2020; Xiao and Kondo, 2020). Therefore, WRF are potentially biodegradable fungi that are seen as a promising bioremediation agent, especially for compounds that are not easily degraded by bacteria.

To date, several genera of fungi have been investigated for their degradation potential of xenobiotics such as aldrin and dieldrin (Huang et al., 2021; Lin et al., 2021). Fragoeiro and Magan (2005) studied the WRF *Phanerochaete chrysosporium* and *Trametes versicolor*, which can completely degrade 5–30 mg⋅L^–1^ of dieldrin within 25 days. The wood-rotting fungal strain YK543, isolated from rotten wood, can degrade approximate 39.1% of dieldrin within 30 days (Fragoeiro and Magan, 2005; Kamei et al., 2010). Xiao also found three WRF, *Phlebia acanthocystis*, *Phlebia brevispora*, and *Phlebia aurea*. They all removed up to 50% of dieldrin and 90% of aldrin in a low-nitrogen medium within 42 days (Xiao et al., 2011a). However, 42 days is a long time, so it is important to isolate fungi that can rapidly degrade aldrin and dieldrin. For the first time, researchers isolated the marine fungus *Penicillium miczynskii* CBMAI 930, which can convert up to 90% (50 mg⋅L^–1^) of dieldrin within 14 days (Birolli et al., 2015). A more efficient WRF fungus, *Pleurotus ostreatus*, was isolated, which completely eliminated aldrin from potato dextrose broth (PDB) medium within 14 days (Purnomo et al., 2017). It also eliminated 18% of the dieldrin in a PDB medium within 14 days.

A strain of *Mucor racemosus* DDF isolated from endosulfan-contaminated soil was able to degrade dieldrin over a wide pH range (Kataoka et al., 2010). The DDF strain is able to grow rapidly in soil compared to white rot fungi, which is an advantage. In addition, *M. racemosus* DDF degrades not only dieldrin, but also many organochlorine pesticides like DDT and endosulfan. Therefore, *M. racemosus* DDF is a potential bioremediation microorganism. This study also showed that endosulfan-contaminated soil is a potential way to isolate dieldrin-degrading bacteria. In addition, dieldrin-degrading fungi such as *Trichoderma viride*, *Mucor alternans*, and *Pleurotus ostreatus* have been isolated by previous researchers, and these fungi can be used as potential microorganisms for bioremediation (Matsumura and Bous, 1967; Purnomo et al., 2017; Zhao et al., 2017). Fungi are more suitable candidates for the bioremediation of dieldrin than bacteria because fungi can degrade these chemicals in a short time with complete mineralization.

#### Degradation of Aldrin/Dieldrin by a Microbial Consortium

Compared with single-bacterium degradation of compounds, microbial consortium degradation has some advantages. Microbial consortium cultures can break down complex mixtures with different microorganisms, increasing the degradation capacity and efficiency (Meyer-Cifuentes et al., 2020; Gielnik et al., 2021; Miao et al., 2021). To date, several microbial consortia have been used to test the degradation of aldrin/dieldrin. The microbial consortium of *Clostridium bifermentans*, *Clostridium glovolium*, and other *Clostridium* sp. is the most efficient, with a degradation rate of 96% of 10 mg⋅L^–1^ dieldrin within 7 days (Maule et al., 1987). Another consortium, containing 11 morphologically identical anaerobic microorganisms, is also very efficient and degrades 75.6% of dieldrin (100 mg⋅L^–1^) and 65.4% of aldrin (100 mg⋅L^–1^) within 2 weeks (Watanabe and Yoshikawa, 2008). However, microbial consortium is not always efficient, probably because of the antagonism. A microbial consortium can transform 88% of dieldrin (9 mg⋅L^–1^) within 3 months, and 70% of aldrin (7 mg⋅L^–1^) within 160 days (Baczynski et al., 2004). A mixed indigenous microorganism containing *Acidaminobacter*, *Clostridium*, and an uncultured bacterial group can degrade low concentrations of dieldrin (from 0.5 to 10 μg⋅mL^–1^) (Chiu et al., 2005). According to these results, the use of a microbial consortium is an effective way to achieve aldrin/dieldrin degradation.

#### Degradation Pathways of Aldrin or Dieldrin

**FIGURE 2:**
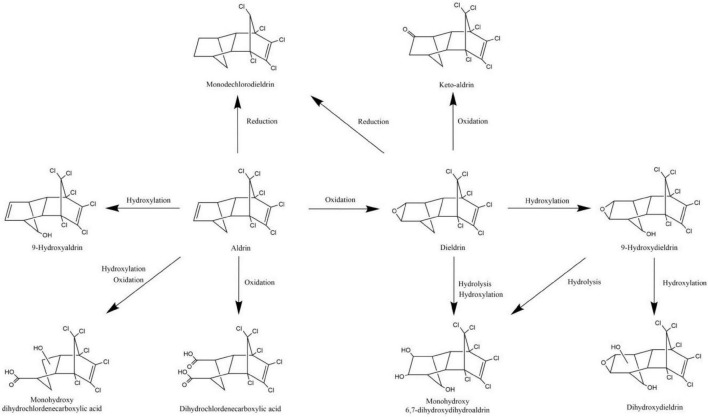
Biodegradation pathways of aldrin and dieldrin (Bandala et al., 2006; Sakakibara et al., 2011; Xiao et al., 2011a).

The degradation pathways of aldrin and dieldrin found in previous research are shown in Figure 2. Dieldrin is often found as a metabolite of aldrin, and aldrin-degrading microorganisms are usually able to degrade dieldrin. The degradation of aldrin can be divided into three types: the oxidation pathway, the reduction pathway, and the hydroxylation pathway (Bandala et al., 2006; Sakakibara et al., 2011; Xiao et al., 2011a). In the oxidation degradation pathway of aldrin, dieldrin is the main metabolite and has a stable structure. When the non-chlorinated double bond is epoxidized in the initial metabolism, aldrin can be converted to dieldrin. When the non-chlorinated double bond is oxidized, aldrin is metabolized by microorganisms to dihydrochlordenedicarboxylic acid and monohydroxy dihydrochlordenedicarboxylic acid. In the reductive degradation pathway, aldrin reacts to form monochlorodieldrin. For highly chlorinated organic compounds, dechlorination reactions are particularly interesting because they usually produce lower toxic metabolites or are readily degradable. When methylene is hydroxylated, aldrin forms 9-hydroxyaldrin.

Degradation of dieldrin includes four pathways: oxidation, reduction, hydroxylation, and hydrolysis (Bandala et al., 2006; Sakakibara et al., 2011; Xiao et al., 2011a). In the presence of microorganisms, dieldrin is oxidized to keto-aldrin or reduced to monochlorodieldrin, which is the same as the reduction product of aldrin. Dieldrin is hydrolyzed to monohydroxy 6, 7-dihydroxydihydroaldrin or 9-hydroxydieldrin. 9-Hydroxydifferent can be further hydrolyzed to 6, 7-dihydroxydihydroaldrin or dihydroxydieldrin, both of which are highly water-soluble (Bandala et al., 2006; Sakakibara et al., 2011).

Based on this degradation pathway, it is inferred that oxidative degradation and reductive dechlorination are important degradation mechanisms of aldrin and dieldrin (Sakakibara et al., 2011). However, neither dieldrin nor aldrin has been documented for the eventual mineralization. In order to further promote the development of biodegradation of aldrin and dieldrin, it is necessary to better understand the degradation mechanism.

#### Gene(s) Involved in the Degradation of Aldrin and Dieldrin

The gene identified as having a high level of resistance to dieldrin (*Rdl*) was first identified in mutant *Drosophila*, where the single amino acid Ala at position 302 was replaced by Ser/Gly (Ffrench-Constant et al., 1993). The SCD strain is resistant to chemical insecticide and the *Bacillus thuringiensis* toxin. When researchers knocked out the *HaRdl-1* gene in the SCD strain, they found that it was more resistant to dieldrin, but when they knocked out the *HaRdl-2* gene in the SCD strain, it was more sensitive to dieldrin. Therefore, it can be speculated that SCD strains develop target-based resistance to dieldrin through a loss of function mutation of *HaRdl-1* or enhancement of expression of *HaRdl-2* (Wang et al., 2020). The *Rdl* gene is present in most types of organisms that feed on crops, while those that do not feed on crops apparently have no resistance. Mutations in *Rdl* occur once or in large numbers, depending on the population biology of the insects. The *Rdl* sites are large (over 50 kb in size) and the alternative splicing is complex.

When the gene at the *Rdl* locus is deleted, mutated insects cannot survive (Ffrench-Constant et al., 2000). In general, most insect species contain only one *Rdl* homologous sequence, such as the Red flour beetle, whiteflies, the coffee berry borer, and *Drosophila* species (Ffrench-Constant et al., 2000). However, pea aphids have two copies of *Rdl* (*Rdl1* and *Rdl2*), which are likely derived from recent gene duplicates (Del Villar and Jones, 2018). *Chilo suppressalis* contains two subtypes of cDNA of the RDL subunit (*CSRDL1A* and *CSRDL2S*). Although *CSRDL2S* expression is twice as high as *CSRDL1A* at all growth stages, they have similar expression patterns (Sheng et al., 2018). G-aminobutyric acid (GABA), a gated chloride ion channel, is an important inhibitory neurotransmitter in the animal nervous system. As a model insect of Lepidoptera, the silkworm (*Bombyx mori*) has the largest known insect GABA gene family in the world, including three *Rdls* (*Rdl1*, *Rdl2*, and *Rdl3*). The three *Rdls* subunits may be generated by two repeated events, among which *Rdl1* gene has an RNA editing site, and *Rdl1* and *Rdl3* can perform alternative splicing (Yu et al., 2010).

In nematode worms, scientists identified a new cyst-ring GABA receptor subcluster (*HCo-LGC-38*) that is moderately sensitive to fipronil and dieldrin (Siddiqui et al., 2012). *S2-RDL^A302S^* of the *Drosophila melanogaster* cell line increased its resistance to dieldrin (Buckingham et al., 1996). The expression of the heterologous *bph* gene in *F113pcb* can enable transgenic microorganisms in the rhizosphere to utilize biphenyl as the sole carbon source (Brazil et al., 1995).

Worryingly, *Rdl* sites have also been shown to be resistant to new insecticides such as fipronil; this cross-resistance must be overcome or avoided (Ffrench-Constant et al., 2000). Lindane and cyclodiene pesticides are the first generation of non-competitive antagonists (NCAs) that enable insects to produce *Rdl* GABA receptors. Fipronil is a second-generation NCA, and extra attention needs to be paid to fipronil-resistant insects to prevent further agricultural losses (Nakao, 2017). Alanine 302 of exon 7 of the *Rdl* was replaced by a serine in the cat flea (*Ctenocephalides felis*) population, thus developing resistance to fipronil (Daborn et al., 2004). Alanine 302 of exon 7 of *Rdl* was replaced by a serine in the cat flea population, leading to resistance to fipronil (Daborn et al., 2004). However, the A302S mutation of *Rdl* has limited cross-resistance to fipronil and is not detected in field populations of the American housefly (Gao et al., 2007). These are grouped in Table 2 for easy reference.

**TABLE 2:** Genes that act on aldrin and dieldrin.

No.	Genes	Resources	Comments	References
	*Rdl*	Mutant *Drosophila*	The single amino acid Ala at position 302 was replaced by Ser/Gly	Ffrench-Constant et al., 1993
	*Rdl*	The Red flour beetle, whiteflies, the coffee berry borer, and *Drosophila* species	Only one RDL homologous sequence	Ffrench-Constant et al., 2000
	*Rdl1* and *Rdl2*	Pea aphids	Derived from recent gene duplicates	Del Villar and Jones, 2018
	*Rdl1*, *Rdl2*, and *Rdl3*	Silkworm	Generated by two repeated events; the *Rdl1* gene has an RNA editing site, and *Rdl1* and *Rdl3* can perform alternative splicing.	Yu et al., 2010
	*Rdl*	Cat fleas (*Ctenocephalides felis*)	Also resistant to fipronil	Daborn et al., 2004
	*CSRDL1A* and *CSRDL2S*	*Chilo suppressalis*	CSRDL2S expression is twice as high as CSRDL1A at all growth stages; they have similar expression patterns	Sheng et al., 2018
	*Bph*	Engineered transposon TnPCB	Utilizes biphenyl as the sole carbon source	Brazil et al., 1995
	*HCo-LGC-38*	Nematode worms	Moderately sensitive to fipronil and dieldrin	Siddiqui et al., 2012
	*S2-RDL^A302S^*	*Drosophila melanogaster* cell line	Increased resistance to dieldrin	Buckingham et al., 1996
	*HaRdl-1*/*HaRdl-2*	The SCD strain	Loss of function mutation of HaRdl-1 or enhancement of expression of HaRdl-2	Wang et al., 2020

## Conclusion and Future Prospects

Aldrin and dieldrin are widely used for their insecticidal activity, but are restricted because of the toxic effect on the environment. Both insecticides have been used for a long time, and their toxic effects on non-target organisms cannot be ignored as aldrin and dieldrin accumulate in the environment and in food chains. Residual aldrin and dieldrin are toxic to a variety of animals, including humans, dogs, fish, shrimp, and other non-target organisms. Therefore, it is necessary to develop efficient, economical, and environmentally friendly technologies for the degradation of aldrin and dieldrin. Microorganisms have the potential to detoxify exogenous compounds easily through several metabolic pathways, and detoxification by indigenous microorganisms is recognized as the most promising remediation approach. A microbial consortium has reported as having superior performance over a pure culture. The reason for the improved performance is the reduction of the metabolic burden. However, the published literature on the microbial remediation of aldrin/dieldrin-contaminated sites remains inadequate. In addition, the development of biodegradable genes/enzymes and degradation microorganisms must be an ongoing project in the future. In recent years, the biodegradation of pesticides based on omics technology has provided a new direction for the bioremediation of environmental pollution by pesticides. Omics-based evaluations could explore in more depth the mechanisms involved in the bioremediation of these xenobiotics.

## Author Contributions

SC conceived of the presented idea. SP and ZL contributed to the writing and prepared the figures and tables. JL, YZ, SM, PB, and SC participated in revising the manuscript. All authors approved it for publication.

## Conflict of Interest

The authors declare that the research was conducted in the absence of any commercial or financial relationships that could be construed as a potential conflict of interest.

## Publisher’s Note

All claims expressed in this article are solely those of the authors and do not necessarily represent those of their affiliated organizations, or those of the publisher, the editors and the reviewers. Any product that may be evaluated in this article, or claim that may be made by its manufacturer, is not guaranteed or endorsed by the publisher.
